# Chain Breakage in the Supercooled Liquid - Liquid Transition and Re-entry of the λ-transition in Sulfur

**DOI:** 10.1038/s41598-018-22775-y

**Published:** 2018-03-14

**Authors:** Linji Zhang, Yang Ren, Xiuru Liu, Fei Han, Kenneth Evans-Lutterodt, Hongyan Wang, Yali He, Junlong Wang, Yong Zhao, Wenge Yang

**Affiliations:** 1 Laboratory of High Pressure Physics, School of Materials Science and Engineering, Southwest Jiaotong University, Chengdu, 610031 China; 2 Center for High Pressure Science and Technology Advanced Research, 1690 Cailun Road, Shanghai, 201203 China; 3 X-ray Science Division, Argonne National Laboratory, 9700 S. Cass Avenue, Argonne, Illinois 60439 USA; 4 HPSynC, Geophysical Laboratory, Carnegie Institution of Washington, 9700 S. Cass Avenue, Argonne, Illinois 60439 USA; 5 Center for the Study of Matter at Extreme Conditions, Department of Mechanical and Materials Engineering, Florida International University, 1930 SW 145th, Avenue Miramar, FL 33027 USA; 6 Photon Sciences Directorate, Brookhaven National Laboratory, Upton, Shirley, New York 11973 USA

## Abstract

Amorphous sulfur was prepared by rapid compression of liquid sulfur at temperatures above the λ-transition for to preserve the high-temperature liquid structure. We conducted synchrotron high-energy X-ray diffraction and Raman spectroscopy to diagnose the structural evolution of amorphous sulfur from room temperature to post-λ-transition temperature. Discontinuous changes of the first and second peaks in atomic pair-distribution-function, *g*(*r*), were observed during the transition from amorphous to liquid sulfur. The average first-neighbor coordination numbers showed an abrupt drop from 1.92 to 1.81. The evolution of the chain length clearly shows that the transition was accompanied by polymeric chains breaking. Furthermore, a re-entry of the λ-transition structure was involved in the heating process. The amorphous sulfur, which inherits the post-λ-transition structure from its parent melts, transformed to the pre-λ-transition liquid structure at around 391 K. Upon further heating, the pre-λ-transition liquid transformed to a post-λ-transition structure through the well-known λ-transition process. This discovery offers a new perspective on amorphous sulfur’s structural inheritance from its parent liquid and has implications for understanding the structure, evolution and properties of amorphous sulfur and its liquids.

## Introduction

Melting is one of the fundamental phenomena in condensed matter. Compared to melting in the crystalline phase, knowledge of melting in various amorphous materials remains very limited since most of amorphous materials crystallize before the melting point during its the heating process^1^. Amorphous material is regarded as an amorphous solid before the glass transition^2^. After the glass transition, amorphous material is generally referred to as a supercooled liquid up to the melting temperature^2^. Thus, the melting process from an amorphous state to liquid state is strictly a supercooled liquid-liquid transition^2^. As the critical cooling rate is a key parameter of normal amorphous materials fabrication, the critical heating rate is extremely crucial for the direct melting in amorphous materials. Direct melting to a liquid state can only be done without a crystallization process only when the experimental heating rate is above the critical value. Johnson *et al*. reported that at a very high heating rate of 10^6^ K/s, the crystallization was can be avoided before melting in metallic glass^3^. It is reasonable to believe that this direct melting process can be observed in many amorphous substances by using such a high heating rate. For some systems with high glass forming ability, e.g., rosin, their amorphous solids normally have relatively low critical heating rates, normally; and they may directly transfer from their amorphous state into a liquid state^4^. For many other amorphous substances, especially for amorphous elements, studies of their melting behaviors are still needed.

Sulfur is an abundant element in the Earth. It has a complicated phase diagram^5,6^. Poly(a)morphism has been found in sulfur crystal states and liquid states, and the transitions among these phases are closely related to the structures and contents of complicated clusters^5–9^. Studies of the amorphous sulfur are expected to be interesting and complicated because it may also experience sulfur cluster structural transitions. Sanloup *et al*. observed amorphous sulfur by pressure-induced amorphization from crystal sulfur at 40–175 K and 50–100 GPa^10^. Furthermore, a polyamorphic transition was observed in this amorphous sulfur, which was explained as a close analogy with the phase transition of crystal S-III to S-IV, which is associated with the structural transformation of sulfur clusters^10^. However, this amorphous sulfur, as well as the amorphous sulfur obtained from rapidly quenching of liquid sulfur, were unstable and recrystallized very easily at low temperature (e.g. above 175 K)^10^ and room temperature^11,12^, respectively. Sulfur’s extremely high crystallization tendency limits these explorations of phase transitions, especially the direct transition from amorphous sulfur to liquid sulfur. Pressure-induced rapid solidification from liquid provides an alternative way of preparing amorphous materials^13^. The amorphous sulfur thus obtained exhibited relatively high thermal stability^14,15^. At room temperature, it takes about 75 mins to start crystallization^14^. At 269 K in the fridge, above its glass transition temperature *T*_*g*_, the amorphous sulfur can be kept for over one month without any detectable crystallization^15^. In this letter, we investigated the structural evolution of amorphous sulfur from room temperature to post-λ-transition temperature by synchrotron high-energy X-ray diffraction, Raman spectroscopy and Differential Scanning Calorimetry (DSC). Raman spectra and high-resolution pair distribution functions provide detailed information on the evolution of the molecular clusters and short-range structure of amorphous sulfur over a temperature range of 302 K to 445 K.

## Results

**Figure 1:**
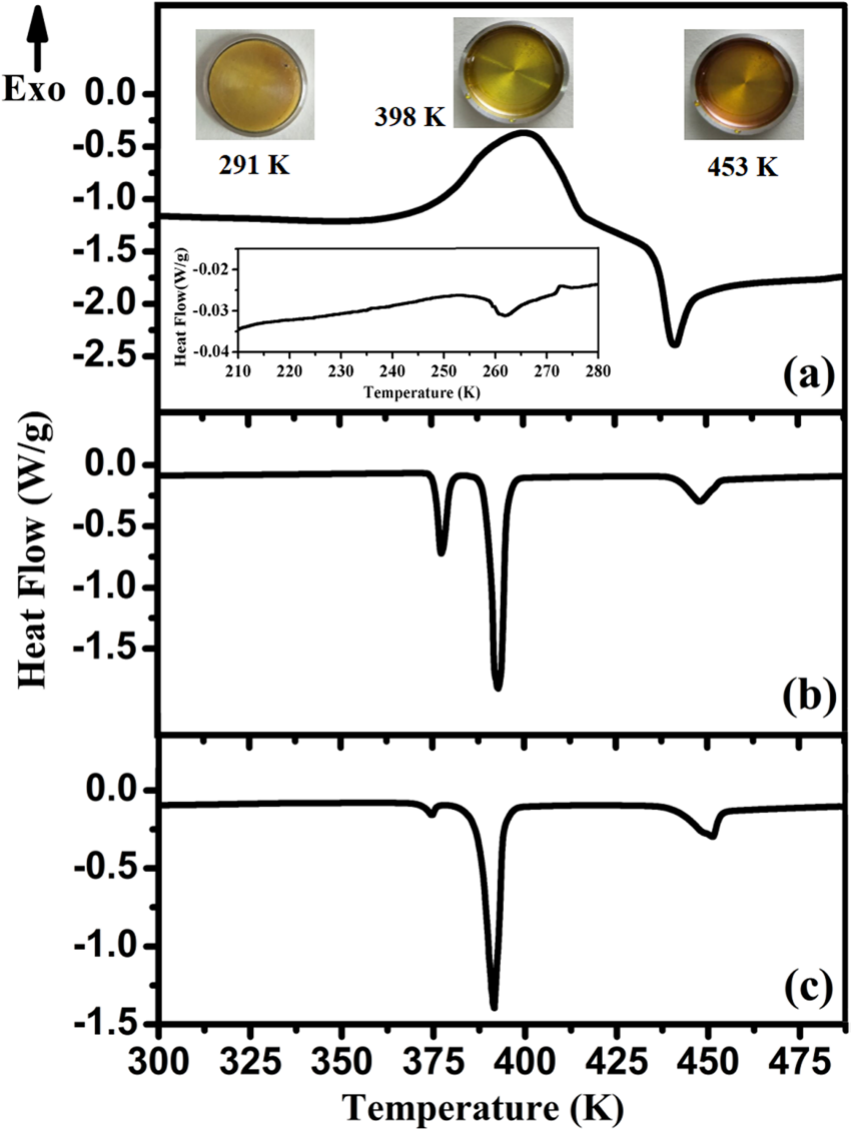
The DSC curves during the 10 K/min heating experiments of (**a**) amorphous sulfur in this work; (**b**) crystal sulfur; (**c**) quenched amorphous sulfur. The inset heat flow curve in Fig. 1(a) is the DSC curve of amorphous sulfur in range of 210–280 K. The inset photos in Fig. 1(a) are the pictures of amorphous sulfur at 291 K, 398 K, and 453 K, which show the changes of sample color, i.e., from dark yellow to light yellow then to dark yellow.

**Figure 2:**
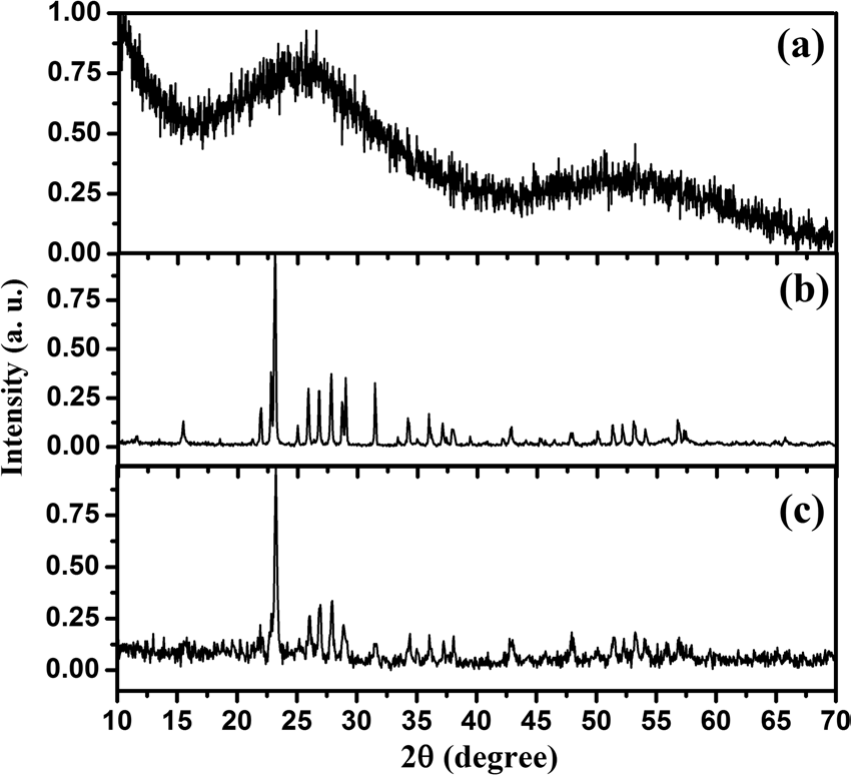
Room temperature XRD patterns of (**a**) amorphous sulfur in this work; (**b**) crystalline sulfur; (**c**) quenched amorphous sulfur, which indicates it crystallized.

Figure 1(a) shows the DSC curve of amorphous sulfur in this work. The inset figure in Fig. 1(a) indicates the glass transition of amorphous sulfur at around 262 K. Therefore at room temperature, the amorphous sulfur in this work is in supercooled liquid state. The DSC curve in Fig. 1(a) displays an exothermic peak at ~400 K and an endothermic peak at ~440 K. The exothermic peak corresponds to the transition from amorphous sulfur to liquid sulfur, while the endothermic peak refers to the λ-transition of liquid sulfur^16^. DSC curves of the crystal sulfur and quenched amorphous sulfur (i.e. rapidly quenched from liquid sulfur to liquid nitrogen temperature) in the range of 300 K-490 K are shown in Fig. 1(b) and Fig. 1(c) for comparison. The melting curve of crystal sulfur contains two sharp endothermic peaks at ~377 K and ~393 K, corresponding to the transition from orthorhombic sulfur (α-S) to monoclinic sulfur (β-S) and the melting of β-S, respectively^6^. Then, the λ-transition of liquid sulfur occurs at 442 K. The quenched amorphous sulfur shows a similar DSC profile to crystal sulfur, indicating it crystallized. For comparison, we conducted the X-ray diffraction on the crystal sulfur, quenched amorphous sulfur and the amorphous sulfur in this work. The results are shown in Fig. 2. The sharp diffraction peaks in Fig. 2(c) indicate that quenched amorphous sulfur transformed to α-S soon at room temperature. The extremely high crystallization tendency has been studied intensively^11,17^. Comparing the thermal behaviors of the three samples, it can be seen that only the amorphous sulfur prepared by rapid compression displayed a direct transition to liquid sulfur. Interestingly, it was associated with an exothermic process. To evaluate the possible changes in the amorphous structure, we conducted synchrotron high-energy X-ray diffraction measurements in the range of 302 K-445 K.

### High-energy XRD analysis of amorphous sulfur

**Figure 3:**
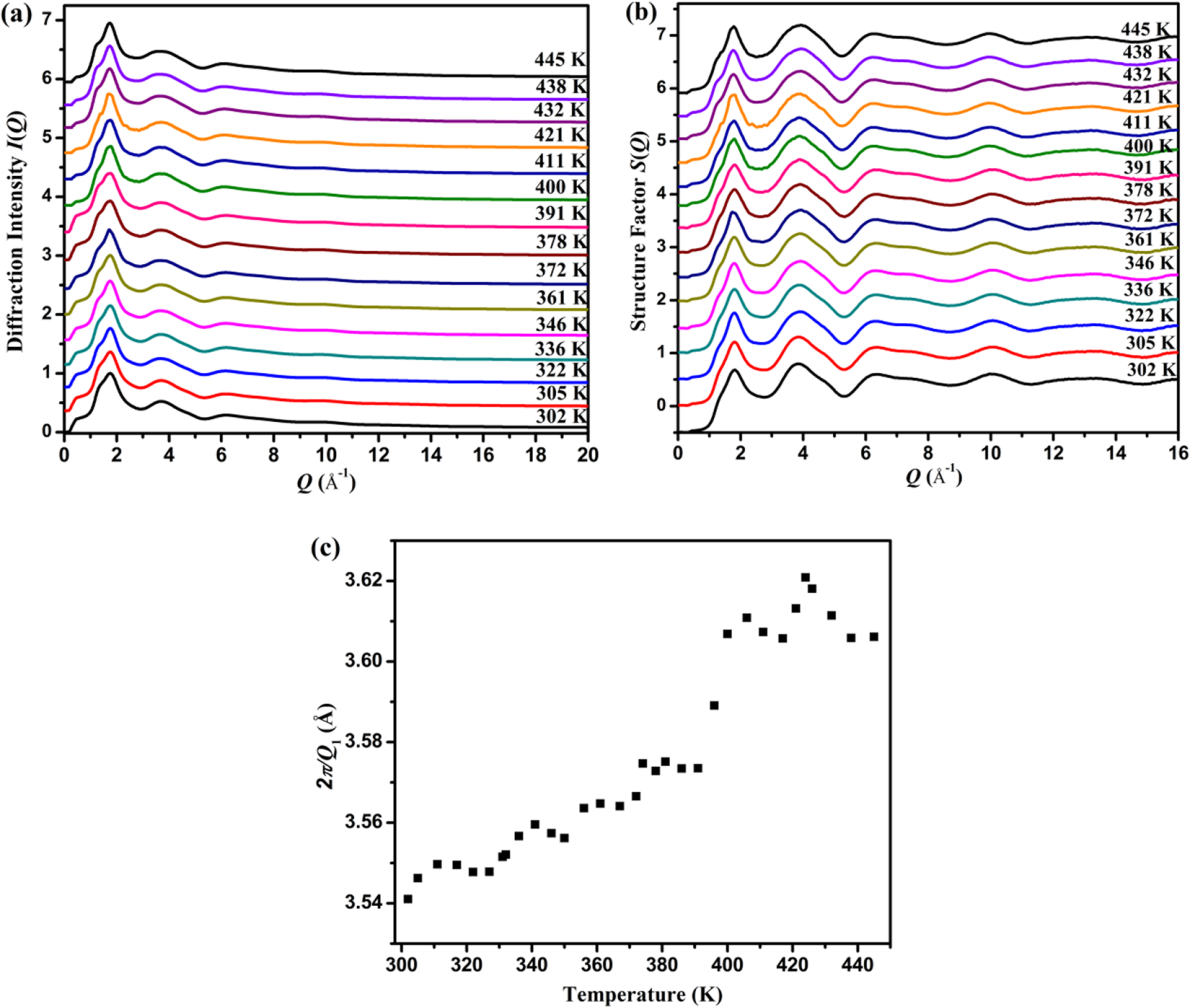
Structural evolution investigated by high energy X-ray diffraction at various temperatures. (**a**) Diffraction intensity profiles *I*(*Q*) at selected temperatures; (**b**) Structure factors *S*(*Q*) at selected temperatures; (**c**) The inverse FSDP position 2π/*Q*_1_ of amorphous sulfur as a function of temperature.

To understand the DSC profile, we have conducted the high-energy XRD to obtain the pair distribution function (PDF) of sulfur as a function of temperature. Figure 3(a) shows the series of XRD patterns taken between 302 K and 445 K. No sharp crystalline diffraction peaks were observed, indicating a direct transition from amorphous sulfur to liquid sulfur. The intensity profiles *I*(*Q*) were treated with sample absorption, inelastic scattering, and polarization correction to obtain the corresponding structure factor *S*(*Q*), as shown in Fig. 3(b). At 302 K, the first maxima of *S*(*Q*) consists of a shoulder at ~1.20 Å^−1^ and main peak at *Q*_1_ = 1.77 Å^−1^. The second peak is located at *Q*_2_ = 3.88 Å^−1^. The first sharp diffraction peak (FSDP) at *Q*_1_ carries significant information about the medium-range ordering^18,19^, providing statistical information on the average inter-atomic spacing *d* according to the well-known Ehrenfest relationship, i.e., $d\propto (1/{Q}_{1})$. The inverse FSDP position 2π/*Q*_1_ correlates with the volume of amorphous materials with a power law function^20^, and can be conveniently used to estimate the relative volume change. Figure 3(c) plots the temperature dependence of 2π/*Q*_1_. Before 391 K, the 2π/*Q*_1_ increases with temperature, as expected from the thermally induced volume expansion in amorphous sulfur. Then, an abrupt jump in 2π/*Q*_1_ is observed at around 391 K. We speculate that the melting process starts at this temperature and the abrupt expansion of volume probably stems from the structural transformation. Above 400 K, the increase of 2π/*Q*_1_ with increasing temperature became gradual within a few degrees, and a relatively small jump in 2π/*Q*_1_ occurs at around 411 K. Then, above 438 K, the increase of 2π/*Q*_1_ with increasing temperature becomes gradual again, and we think that the amorphous sulfur turns into a fully molten state. The melting temperature of 391 K is close to the reported melting temperature of 392 K from crystalline sulfur^6,21^.

**Figure 4:**
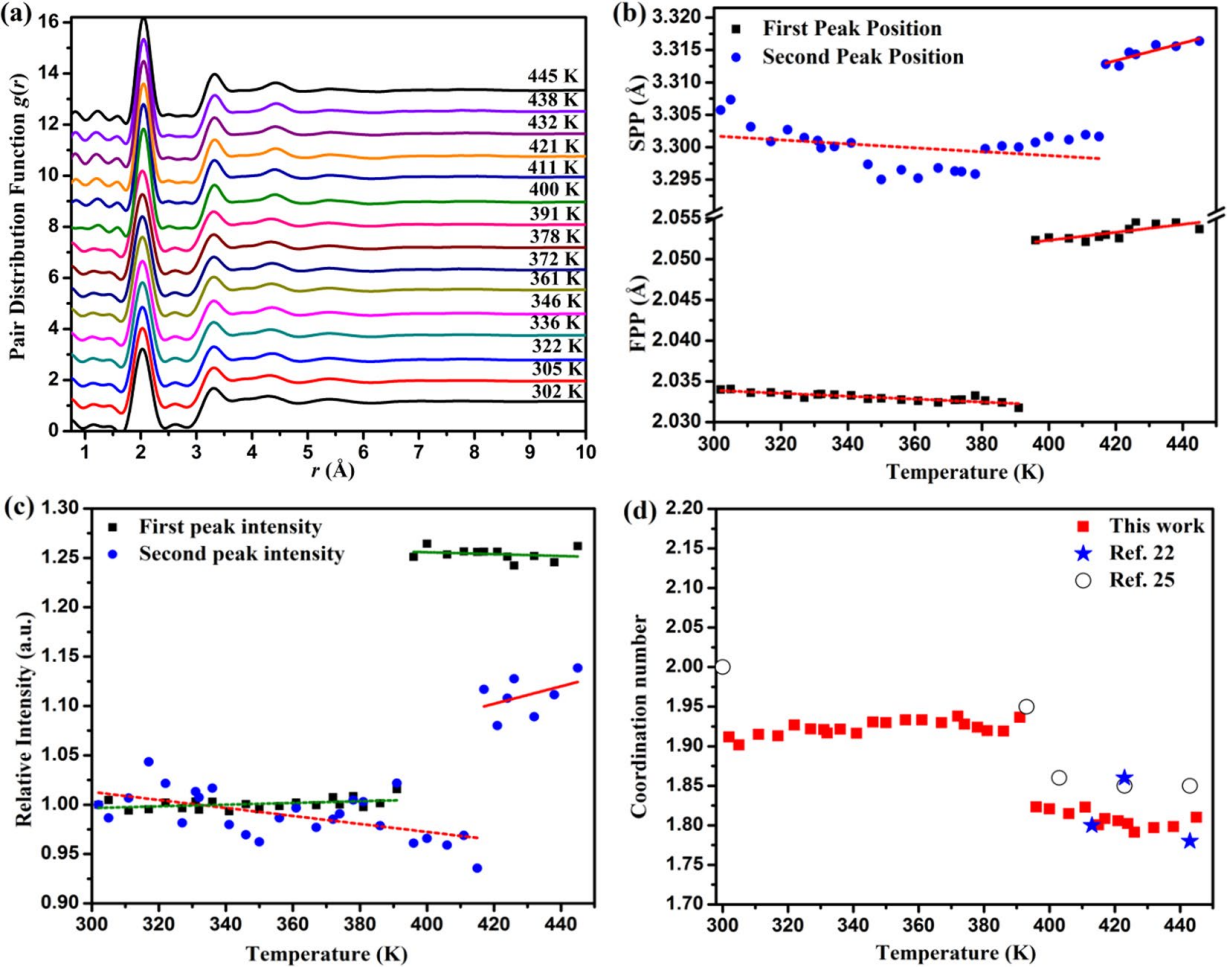
Structural evolution of amorphous sulfur in real space. (**a**) Pair distribution function *g*(*r*) at selected temperatures; (**b**) The position of the first and second peak of *g*(*r*) at a temperature range 302 K to 445 K; (**c**) The peak intensity of the first and second peak in *g*(*r*) as a function of temperature. The position and intensity in the *g*(*r*) peak were obtained by a Gaussian fitting; (**d**) Coordination numbers in the first nearest-neighbor shell of amorphous sulfur at different temperatures. Data of crystal sulfur from ref.^22^ and ref.^25^ are shown for comparison.

The data *S*(*Q*) was then Fourier transformed to the pair distribution function *g*(*r*) (see Fig. 4(a)), which revealed information on the short-range order of amorphous sulfur. At 302 K, the first peak of *g*(*r*) was located at *r*_1_ = 2.034 Å. The distance *r*_2_ of the next-nearest neighbors was centered around 3.306 Å, and the third peak appeared around 4.292 Å. The corresponding three *g*(*r*) peaks of the parent liquid were located at ~2.05 Å, ~3.31 Å, and ~4.45 Å, respectively^22^. The obvious shortening of the interatomic distance in amorphous sulfur is ascribed to the volume contraction during the solidification of the parent liquid and temperature difference. In Fig. 4(a), no abrupt changes of the *g*(*r*) curve shape were observed with increasing temperature. However, we did observe minor changes in the strengthening of the two weak humps before the first peak and the weakening of the hump between the first and second peaks with increasing temperature and their discontinuous change seemed to appear at 391 K. Figure 4(b) and (c) display the dependence of the position and intensity of the first and second peaks on temperature. For the first peak, an abrupt jump occurred at 391 K; its position shifted from 2.034 Å to 2.053 Å and relative intensity increased by 23.23%. For the second peak, the abrupt jump occurred at 411 K, and its position shifted from 3.302 Å to 3.313 Å with relative intensity dropping by 13.71%. The two jumps of volume in Fig. 3(c) seem related to the two discontinuous changes in the first and second nearest neighbor peaks of *g*(*r*). That is to say that the abrupt expansion of volume is largely related to short-range order changes.

To reveal structural information on the first nearest-neighbor shells of the amorphous sulfur, we calculated the first nearest-neighbor coordination numbers (*CN*) *n*_1_. Based on the concept that the coordination shell is symmetrical around a radius, which defines the maximum in the *r*^2^*g*(*r*) curve, the right-hand side of the peak is made symmetrical with that on the left. The *CN* is determined by the integration:

1 $$2{\int }_{{r}_{0}}^{{r}_{\max }}4\pi {\rho }_{0}[{r}^{2}g(r)]dr$$

where *r*_0_ and *r*_max_ is the left-hand edge and peak position of the peak, respectively, in the *r*^2^*g*(*r*) curve, and *ρ*_0_ is the number density of atoms^23,24^. The coordination numbers *n*_1_ thus obtained as a function of temperature is shown in Fig. 4(d). It is found that the *n*_1_ of amorphous sulfur is less than 2 for all temperatures, which indicates that the first peak observed at *r* = 2.034 Å contains on average less than two atoms within the sulfur molecules for all temperatures. For the models such as the S_8_ rings and long freely rotating chains, the *n*_1_ should be 2 at 2.06 Å^22^. This suggests that the local chain and ring structures broke up for all temperatures in amorphous sulfur. No abrupt change of *n*_1_ accompanying the temperature increment was observed before 391 K, which indicates that the short-range order remained before melting. An abrupt drop of *n*_1_ from ~1.92 to ~1.81 then clearly appeared at around 391 K, implying a sulfur cluster structural change during the melting process. Crystal sulfur data from ref.^22^ and ref.^25^ are shown in comparison with that of the amorphous sulfur. At room temperature, the *n*_1_ of crystal sulfur is 2, which corresponds to the structure of the S_8_ rings^25^. After melting, the *n*_1_ dropped to around 1.81^22^, which matches experimental observation in this work very well. From the *n*_1_ value, we can say that after melting, the amorphous sulfur returns to a similar liquid melted from crystal sulfur.

To check the identity of the liquid sulfur from the amorphous sulfur melting and that from the crystal sulfur melting, we compared the peak positions of *g*(*r*) and coordination numbers from this work with that from earlier HEXRD measurements reported in ref.^25^, as listed in Table 1. The data in this work at 421 K match those reported in ref.^25^ at 423 K, which indicates that the liquid sulfur structure melted from the amorphous sulfur or crystal sulfur takes the same form. When the pressure and temperature conditions reach the thermodynamically stable region of the pre-λ-transition liquid, both amorphous sulfur and crystal sulfur transform into the pre-λ-transition liquid. In the pre-λ-transition liquid, the *n*_1_ is around 1.81, which is consistent with earlier studies of liquid sulfur with neutron diffraction techniques^22^. Since *n*_1_ = 1.81 is lower than that the S_8_ rings structure, a conjecture is that the S_8_ ring structure at the pre-λ-transition liquid temperature breaks up at least partially, compared to the crystal structure with 100% S_8_ rings^22^. The results in this work support this assumption. Upon further heating, the λ-transition occurs as expected. The DSC trace of amorphous sulfur in Fig. 1(a) clearly showed the endothermic peak of the λ-transition. In Table 1, the first three nearest neighbor distances (*r*_1_, *r*_2_, *r*_3_ ~ 2.05 Å, 3.32 Å, 4.37 Å) and coordination numbers (*n*_1_, *n*_2_ ~ 1.8, 3.0) at 445 K (above λ-transition temperature) are consistent between our data and that from ref.^25^. This demonstrates that both amorphous sulfur and crystal sulfur become to the post-λ-transition liquid. When evaluating the structural evolution of the starting amorphous sulfur at room temperature from post λ-transition, we note that this process involved a re-entry of the post-λ-transition structure. However, the starting amorphous sulfur has a coordination number around 1.91, which is much larger than that from the post-λ-transition liquid 1.81, we would suggest the short sulfur chains connected to longer chains during the rapid compression period. This partially inherited post-λ-transition structure sample has much higher kinetic energy barrier to transfer to crystalline sulfur compared to amorphous sulfur synthesized by other means, which in turn, makes the amorphous more stable.

**Table 1:** The nearest neighbor positions *r* and coordination numbers *n* for amorphous sulfur and liquid sulfur under different temperatures.

	*T*(K)	*r*_1_(Å)	*r*_2_(Å)	*r*_3_(Å)	*n*_1_	*n*_2_
Amorphous sulfur	302	2.034	3.306	4.292	1.91	3.37
	421	2.053	3.313	4.404	1.81	2.96
	445	2.054	3.316	4.366	1.81	2.99
Liquid sulfur^25^	423	2.050	3.34	4.50	1.85	3.07
	443	2.049	3.33	4.50	1.85	3.05

### Raman Spectroscopy analysis of amorphous sulfur

**Figure 5:**
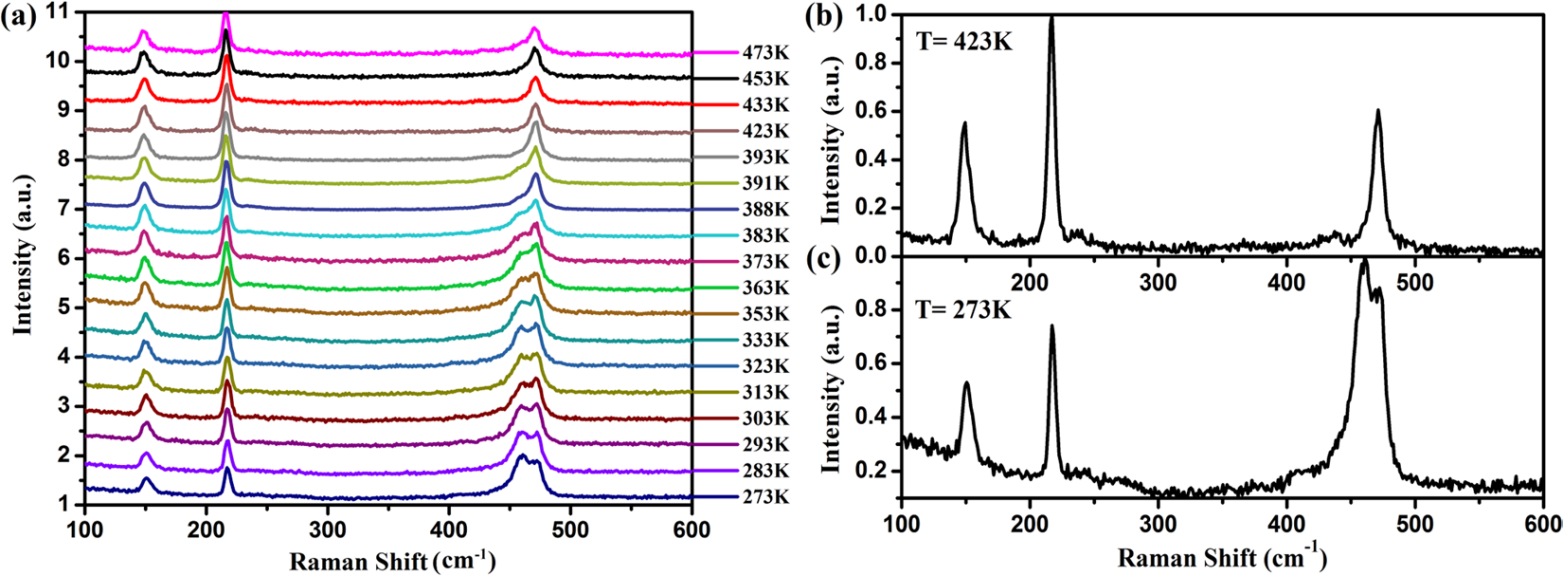
(**a**) Raman spectra of amorphous sulfur at selected temperatures. Raman spectrum of amorphous sulfur at (**b**) 423 K and (**c**) 273 K. The intensities are normalized.

Raman spectroscopy serves as an effective tool to investigate the characteristics of local bonding arrangements, which provides finger prints for chains vs. rings^26,27^. To clarify the structural transition behavior of the sulfur clusters during the melting process in amorphous sulfur, Raman spectra were studied in the range of 273 K–473 K. Figure 5(a) displays the Raman spectra of amorphous sulfur at selected temperatures. Figure 5(b) and (c) show the Raman spectra of the sample at 423 K and 273 K, respectively. The Raman spectrum at 423 K shows a similar pattern to liquid sulfur after crystal melting at 428 K in ref.^27^ which reconfirms that amorphous sulfur melts into pre-λ-transition liquid sulfur. According to earlier references, the Raman peaks located at ~150 cm^−1^and ~220 cm^−1^ are assigned to the anti-symmetric bond-bending and the symmetric bond-bending modes of the S_8_ rings, respectively^27^. The peak located at ~472 cm^−1^is assigned to the symmetric S-S bond-stretching mode of the S_8_ rings^27^. As previously mentioned, the HEXRD and neutron diffraction results suppose that the short chains exist in the liquid sulfur before the λ-transition^22^. Andrikopoulos *et al*. suggested that there are two Raman peaks at ~462 cm^−1^ and ~470 cm^−1^ representing the vibrational modes of the two types of sulfur chains, i.e., a low molecular weight chain species and a high molecular weight chain species^17^. The peak at ~462 cm^−1^ has frequently been assigned to the S-S bond stretching of the long polymeric chains^27^. When the vibrational mode of the short chains locates at ~470 cm^−1^, it is almost indistinguishable from the ~472 cm^−1^ peak of the S_8_ rings due to the overlap of the two peaks and the limitation of the spectroscope’s resolution. Therefore, it is hard to estimate the contents of the short chains from the Raman spectra of liquid sulfur by using the ~470 cm^−1^ peak. In Fig. 5(c), the Raman spectrum at 273 K clearly shows a peak at ~461 cm^−1^, which indicates the existence of polymeric chains in amorphous sulfur. As shown in Fig. 5(a), the 461 cm^−1^ peak as a shoulder of the ~472 cm^−1^ peak existed up to 383 K. However, in Fig. 5(b), the 461 cm^−1^ peak diminished after melting, which suggests that the melting process is associated with the structural transition of the molecular clusters, i.e., the breakage of the polymeric chains.

**Figure 6:**
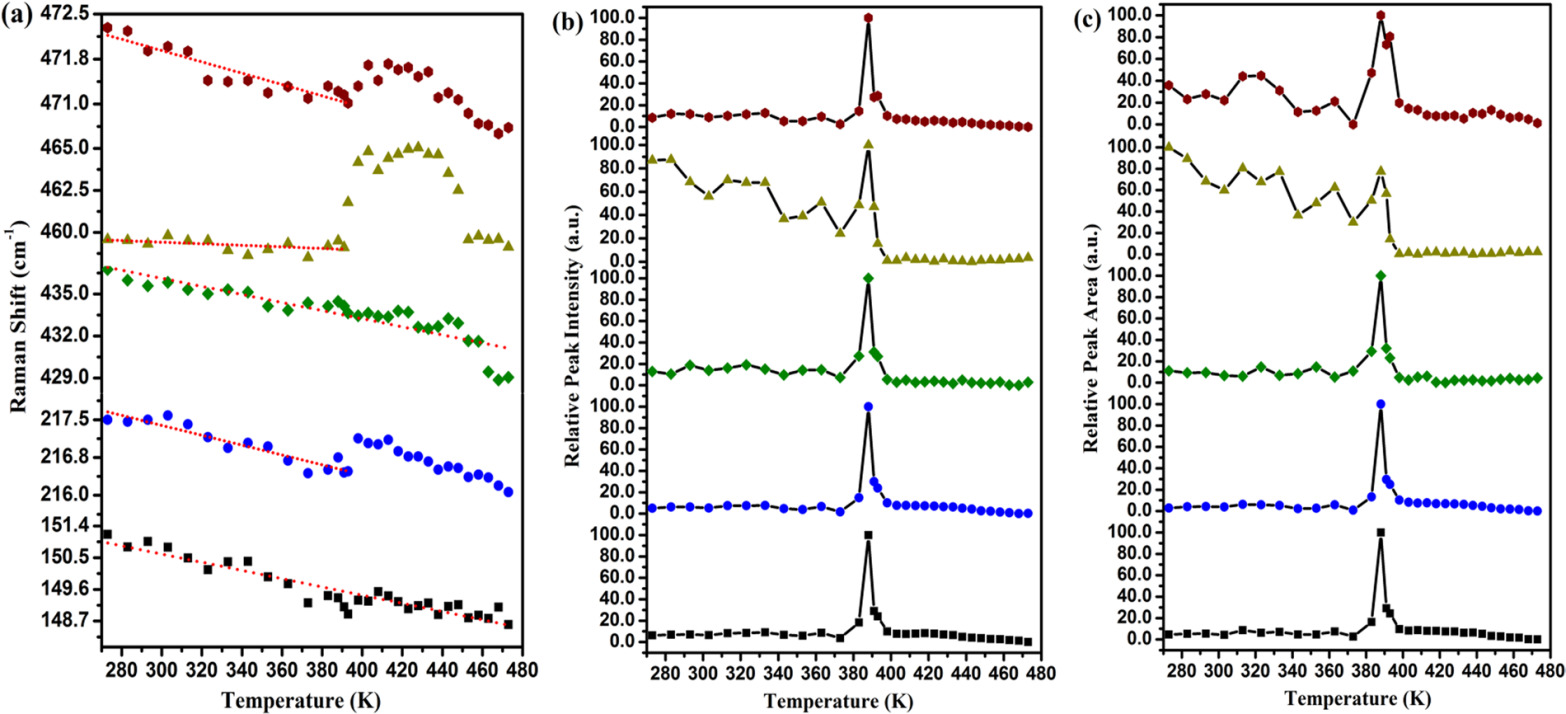
(**a**) Temperature-dependent Raman shifts related to the S_8_ ring and polymeric chain modes; (**b**) The relative intensity evolution of the Raman peaks with increasing temperature; (**c**) The relative area evolution of the Raman peaks with increasing temperature.

Details about the evolution of the position, relative intensity, and area of the Raman peaks with increasing temperature are displayed in Fig. 6. In Fig. 6(a), the peaks located at ~150 cm^−1^, ~220 cm^−1^, ~436 cm^−1^, and ~472 cm^−1^have a continuously softening trend with increasing temperature. The temperature dependence of these vibration modes, i.e., $d\tilde{\upsilon }/dT$ were −0.0110 cm^−1^/K, −0.0104 cm^−1^/K, −0.0272 cm^−1^/K, and −0.0102 cm^−1^/K, respectively. However, we observed minor discontinuous shifts of the peak positions around 0.67 cm^−1^ and 0.62 cm^−1^ for the ~220 cm^−1^ and ~472 cm^−1^modes, respectively, at around 391 K. Although these shifts were within the error bar, these minor discontinuous changes of peak position were probably connected with the melting behavior at around 391 K. The peak located at ~461 cm^−1^ showed a distinctive, very weak downward trend with increasing temperature. The $d\tilde{\upsilon }/dT$ of 461 cm^−1^ peak was −0.0075 cm^−1^/K before 391 K. After 391 K, it shifted backward to a high wavelength by 8.3 cm^−1^ within the temperature range of 20 K. The highest wavelength value was 467.1 cm^−1^ at 411 K and we speculate that it corresponds to the short chains’ mode, which is suggested to be around 470 cm^−1^ in liquid sulfur^17^. It was surprising that the abnormal backward shift of the 461 cm^−1^ mode during melting was related to the polymeric chain breakage. The temperature dependence of the relative intensity and area of all vibration modes is shown in Fig. 6(b) and Fig. 6(c). There was a sudden increase in the intensity and area for all vibration modes at around 391 K, which originates from the increase in the distance between the atoms during the melting process. The ratio of *Φ*(*T*) was employed to evaluate the content of the polymeric chains in sulfur, where *A*^461^ and *A*^472^ denote the integrated area of the peaks located at ~461 cm^−1^ and 472 cm^−1^, respectively.

2 $$\Phi (T)={A}^{461}/({A}^{472}+{A}^{461})$$

**Figure 7:**
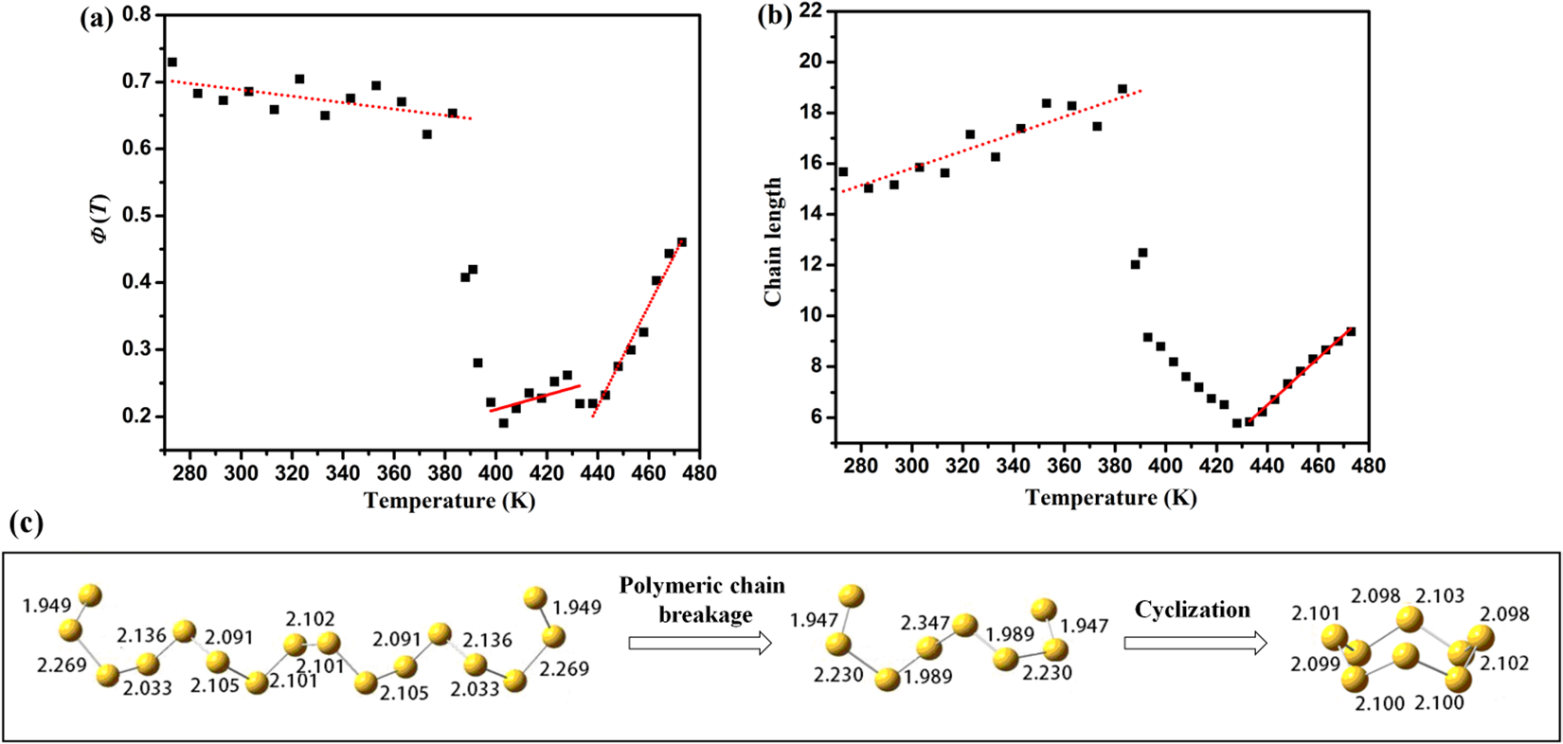
(**a**) Temperature dependence of polymerization *Φ*(*T*) calculated by the area ratio *A*^461^/(*A*^472^ + *A*^461^); (**b**) The average chain length as a function of temperature; (**c**) Three types of sulfur clusters i.e. S_16_ chain (*E* = −6371.02898)_,_ S_8_ chain (*E* = −3185.48365) and S_8_ ring (*E* = −3185.55778) calculated at the B3LYP/6–31 + G* level. The energy *E* is given in Hartree (1 Hartree ≈ 627.51 kcal/mol) and the S-S bond lengths are given in Å.

As shown in Fig. 7(a), the *Φ(T)* was 0.73, i.e., the polymeric content was about 73 wt% at 273 K, inferring that most of the amorphous sulfur content is polymeric chains in this amorphous sulfur. This value is obviously higher than that of the parent liquid, i.e., the *Φ*(*T*) was ~30 wt% at 453 K in Fig. 7(a). From this point of view, we propose that pressure affects the content of the polymeric chains in amorphous sulfur during the fast compression process, which has been reported in our earlier work^28^. The polymeric content decreases in a systematic and directional way at temperatures higher than 391 K. This temperature matches the melting temperature suggested by XRD results. We presume that the linear decrease of *Φ*(*T*) corresponds to the breakage of the polymeric chains during melting. Above 440 K, the *Φ*(*T*) increased gradually with increasing temperature, in agreement with an earlier report that the polymeric content increases above the λ-transition temperature^17,29^.

## Discussion

Based on a simple chain model, we calculated the average chain length in amorphous sulfur before 391 K. The average first neighbor *CN* of a chain structure is 2-2/*N*, where *N* is the length of the chain^9^. The *CN* of polymeric chains was calculated from the average first neighbor *n*_1_ of the sulfur clusters and deducting the contribution of rings by the following relationship.

3 $${n}_{1}=CN\times \Phi +{\Phi }_{ring}\times 2$$

4 $${\Phi }_{ring}=1-\Phi $$

where *Φ* and *Φ*_*ring*_ are the content of polymeric chains (see formula (2)) and rings respectively. The average chain length *N* in amorphous sulfur was then calculated from the *CN* of chains and is shown in Fig. 7(b). The average chain length *N* is ~16 before 383 K. Then the *N* dropped suddenly, which suggests the polymeric chains broke. Assuming that the polymeric chains broke into S_8_ chains whose coordination number is 1.75 and the content of rings remains, the *CN* of residual chains (corresponding to *Φ*) after 391 K was calculated by the following relationship

5 $${n}_{1}=CN\times \Phi +{\Phi }_{ring}\times 2+(1-\Phi -{\Phi }_{ring})\times 1.75$$

The average chain length *N* was then calculated from the *CN*. The *N* is ~8 after 391 K, which means all polymeric chains transformed into S_8_ chains. This result is consistent with the 461 cm^−1^ peak shifting to high wavelength and trending to the Raman mode of the short chains in Fig. 6(a). It is generally accepted that the pre-λ-transition liquid mainly consists of S_8_ rings. We speculated that partial S_8_ short chains afterwards transformed to S_8_ rings in the melting process.

In normal melting process, the clusters depart each other against the Van der Waals interaction among them, which is an endothermic effect. To explain the exothermic effect during melting, three types of sulfur clusters i.e. S_16_ chain, S_8_ chain and S_8_ ring were optimized by using the B3LYP/6–31 + G* density functional method^30^. The B3LYP/6–31 + G* is a combination of the Becke three-parameter exchange functional i.e. B3 and the dynamic correlation functional of Lee, Yang, and Parr i.e. LYP^31,32^. All the computations were performed by the Gaussian09 package^33^. The initial optimized coordinates for each structure are taken from sulfur crystal structures^5^. The stable S_16_ chain, S_8_ chain and S_8_ ring cluster structures and energies are shown in Fig. 7(c). The polymeric chain-ring transition can be described as 1/2S_16_ chain_ → _S_8_ chain_ → _S_8_ ring_._ Breaking up of S_16_ chain to S_8_ chain is endothermic and needs the energy of 316 J/g. Then the S_8_ chain cyclizates to form the S_8_ ring, in which 760 J/g energy will be released. Thus a 444 J/g net energy will be released, which present an exothermic behavior. The exothermic peak in the DSC curve is a comprehensive coupled effect of the polymeric chain breakage, short chain-ring transition and the normal melting process.

The λ-transition occurs at 442 K in the liquid sulfur, which is associated with an abnormal increase of viscosity by 4 orders of magnitude and rapid changes in its optical and thermodynamic properties^34,35^. Although this phenomenon has been explored for more than 150 years, it remains unsolved^35^. The λ-transition has been mostly interpreted as a liquid-liquid transition from an S_8_ ring monomer to a polymeric phase^17,21,29^. On account of the color evolution, the λ-transition was speculated to be related to the electric state transformation of chains^36^. Scopigno *et al*. proposed that the abnormal increase of viscosity is related to structure relaxation of chains^35^. In this work, the re-entry of the λ-transition was achieved by applying pressure and temperature. The DSC curve shows that the amorphous sulfur solidified from the post-λ-transition liquid directly transformed to a pre-λ-transition liquid through an exothermic process. Upon heating, the pre-λ-transition liquid transformed to a post-λ-transition liquid through the endothermic process of the λ-transition. Synchrotron high-energy XRD and Raman spectroscopy confirmed the identity between the liquid sulfur from the amorphous sulfur melting and that from the crystal sulfur melting. In Fig. 1(a), the amorphous sulfur shows a similar color of dark yellow to the post-λ-transition liquid. It was speculated that the amorphous sulfur inherited the electric state of parent liquid. After melting it transformed to a light yellow pre-λ-transition liquid before changing to dark yellow again after the λ-transition. From these points of view, the re-entry of the λ-transition was achieved by applying pressure and temperature.

## Summary

As an elementary substance, amorphous sulfur provides a very valuable subject to study disordered states and amorphous phase transitions. Amorphous sulfur fabricated by the rapid compression of molten sulfur exhibits outstanding thermal stability. This is ascribed to the high polymeric chain content, which is related to the pressure-loading procedure. Nevertheless, as a metastable phase, amorphous sulfur can be transferred to stable crystal sulfur by crystallization. However, crystallization is a time-dependent kinetic process. When choosing a heating rate higher than the critical value, crystallization can be avoided. In these circumstances, we can study the direct transition of amorphous sulfur to liquid sulfur. In this work, we demonstrate the evolution of the short-range order and sulfur cluster structure during the melting amorphous sulfur processing. The change in the first-neighbor coordination numbers indicates that the melting process is associated with the chain breaking. The decline of the bond stretching mode of the polymeric chains at ~461 cm^−1^ in the Raman spectra confirmed the chains broke during the melting. The amorphous sulfur studied in this work partially inherited the polymeric structure of the post-λ-transition parent liquid. At 391 K, this polymeric dominated amorphous phase melted to the pre-λ-transition liquid structure like from crystalline sulfur. The heating process from melting to the λ-transition involved a re-entry of the post-λ-transition structure. This re-entry of the post-λ-transition structure offers a new perspective on amorphous sulfur’s structural inheritance from its parent liquid and has implications for understanding the structure, evolution, and properties of amorphous sulfur and its liquids.

## Methods

The amorphous sulfur sample was prepared using a rapid compression technique. Crystal sulfur (99.999% purity from Shanghai Chem. Co. of China Medicine Group) was heated to 453 K (above the ambient pressure λ-transition temperature) inside a piston-cylinder apparatus, and then rapidly compressed to 2.3 GPa within 20 milliseconds. Details of the amorphous sulfur preparation have been reported elsewhere^28^. The crystal sulfur and quenched amorphous sulfur were also analyzed for comparison. The quenched amorphous sulfur was prepared by rapidly injecting liquid sulfur at 453 K into liquid nitrogen. Differential Scanning Calorimetry (DSC) analysis were conducted on TA-Q1000 and TA-Q2000 instruments with a heating rate of 10 K/min. The weighing and loading to the calorimeter for all samples were conducted at room temperature. The low temperature DSC curve of amorphous sulfur in this work was measured in the range of 210 K-280 K. The X-ray diffraction (XRD) patterns of three samples were taken on diffraction instruments (X’ Pert. PRO. MPD. Philips and DX-2700, China) by using Cu K_α_ radiation at room temperature.

*In situ* high-energy X-ray diffraction (HEXRD) experiments with a focused X-ray beam of about 50 × 50 μm^2^ (FWHM) at a wavelength of 0.11798 Å were performed at beam line 11-ID-C of the Advanced Photon Source (APS) of the Argonne National Laboratory (ANL). The sample was cut into a small stick, around 1 × 1 × 5 mm^3^ and then placed into a cylindrical Polyimide tubing. A cryostream system was used for the temperature dependent measurements. A Perkin Elmer area detector was used to collect the diffraction patterns. The background profile was obtained from an empty tube. The diffraction pattern of the polycrystalline CeO_2_ standard was collected to calibrate the distance between the detector and the sample and detector tilting angles. Two-dimensional X-ray diffraction patterns were integrated using the program FIT2D program^37^. The pair distribution function *g*(*r*) was obtained by a Fourier transformation of *S*(*Q*) using PDFgetX2 software^38^.

The Raman spectra measurements were taken by a back-scattering configuration with a 1.0 cm^−1^ spectral resolution (in-Via, Renishaw). The wavelength of the excitation laser was 633 nm, and the beam size was ~2 × 2 μm^2^. The applied output power of the laser was ~2 mW. A wide-range temperature control device was used (Linkam FRIT600, available temperature range 80 K-873 K) was used. The amorphous sulfur was cut into a ~2.0 mm × 2.0 mm × 0.1 mm slice and placed in a quartz crucible. The accuracy of the temperature controller was around ± 0.1 K. Raman scattering measurements were conducted in the temperature range of 273 K−473 K with a heating rate of 10 K/min and a data collection speed of 20 seconds per acquisition.

### Data availability

All data generated or analyzed during this study are included in this published article.
